# VALIDATION OF MODIFIED SENSE OF CONTROL SCALE AMONG OLDER CANCER PATIENTS

**DOI:** 10.1093/geroni/igae098.2317

**Published:** 2024-12-31

**Authors:** Peiyuan Zhang, Sarah Clem, Ting Guan, Todd Becker, Paul Sacco

**Affiliations:** University of Maryland Baltimore, Baltimore, Maryland, United States; University of Maryland School of Social Work, University of Maryland School of Social Work, Maryland, United States; Syracuse University, Syracuse, New York, United States; Washington University School of Medicine in St. Louis, St. Louis, Missouri, United States; University of Maryland Baltimore, Baltimore, Maryland, United States

## Abstract

Sense of control, defined as the degree to which individuals feel able to influence their behavior and are responsible for the consequences, is a key intervention target for older cancer populations who experience high levels of distress. Despite being widely used, the 10-item Sense of Control Scale (SCS) with two subscales (i.e., Perceived Constraints and Personal Mastery) has not been validated with older cancer populations. This study aimed to examine (1) factorial and convergent validity of SCS and (2) measurement invariance between older cancer and non-cancer populations. Using data (N=2,865) from the most recent wave (2020) of the Health and Retirement Study, we examined factorial validity and measurement invariance using second-order confirmatory factor analysis and multi-group confirmatory factor analysis, respectively. We then assessed convergent validity with depression via Pearson product–moment correlation. SCS had adequate global model fit, χ2(33)=295.43, p<.001 (RMSEA=.08, SRMR=.04, CFI=.94, TLI=.92) and good local fit (rangeλ=.64–.88). Regarding measurement invariance, likelihood chi-square ratio test results showed significant differences in model fit across configural, metric, and scalar models (p<.001), but the magnitude of the change was negligible (ΔCFI<.01). Thus, we concluded that SCS is invariant between older cancer and non-cancer populations. Compared to older non-cancer populations, older cancer populations appeared to have a significantly lower personal mastery (ΔM= –0.12, p=.045) and higher perceived constraints (ΔM= –0.1, p=.047). SCS was negatively correlated with depression (r= –.36, p<.05), showing desirable convergent validity. Overall, the SCS exhibits favorable preliminary psychometric properties with older cancer patients and appears invariant to cancer status.

